# Combining three-dimensional acoustic coring and a convolutional neural network to quantify species contributions to benthic ecosystems

**DOI:** 10.1098/rsos.240042

**Published:** 2024-06-19

**Authors:** Katsunori Mizuno, Kei Terayama, Shoichi Ishida, Jasmin A. Godbold, Martin Solan

**Affiliations:** ^1^ Department of Environment Systems, Graduate School of Frontier Sciences, The University of Tokyo, Kashiwanoha, Kashiwa, Chiba 277-8561, Japan; ^2^ Graduate School of Medical Life Science, Yokohama City University, 1-7-29, Suehiro-cho, Tsurumi-ku, Yokohama 230-0045, Japan; ^3^ School of Ocean and Earth Science, National Oceanography Centre Southampton, University of Southampton, Southampton SO14 3ZH, UK

**Keywords:** convolutional neural network, organism–sediment interactions, bioturbation, ecosystem functioning, species traits, benthic

## Abstract

The seafloor is inhabited by a large number of benthic invertebrates, and their importance in mediating carbon mineralization and biogeochemical cycles is recognized. However, the majority of fauna live below the sediment surface, so most means of survey rely on destructive sampling methods that are limited to documenting species presence rather than event driven activity and functionally important aspects of species behaviour. We have developed and tested a laboratory-based three-dimensional acoustic coring system that is capable of non-invasively visualizing the presence and activity of invertebrates within the sediment matrix. Here, we present reconstructed three-dimensional acoustic images of the sediment profile, with strong backscatter revealing the presence and position of individual benthic organisms. These data were used to train a three-dimensional convolutional neural network model and, using a combination of data augmentation and data correction techniques, we were able to identify individual species with an 88% accuracy. Combining three-dimensional acoustic coring with deep learning forms an effective and non-invasive means of providing detailed mechanistic information of *in situ* species–sediment interactions, opening new opportunities to quantify species-specific contributions to ecosystems.

## 1. Introduction

Coastal and marine waters drive a disproportionately large fraction of productivity [1,2], maintain high levels of biodiversity [2–4] that are of importance to the food web, and for mediating ecosystem properties [5–7], but they are also vulnerable to a range of anthropogenic pressures [8] and to climate change [9]. As human activity increases alongside the expression of climate change, localized modifications to the seafloor become cumulatively significant, resulting in permanent changes to an already modified seabed that can alter ecosystem processes supporting major ecosystem services [10–12]. For the most part, information on changes in benthic community structure and functioning has been obtained either through *ex situ* experimentation [13], with *in situ* observations taken from above the seafloor [14–17], or with destructive sampling devices (grabs, cores, anchor dredges, etc.) [18] that remove species from their three-dimensional habitat. Whilst time-lapse sediment profile imaging techniques (t-SPI) [19] allow observation [20] and quantification [21] of subsurface species behaviour using two-dimensional images and computed tomography allows reconstructions of biogenic features in three dimensions (e.g. burrow networks [22,23]; bioirrigation [24]), there remains a critical need for the development of a non-invasive capability that allows *in situ* observation and quantification of sediment infaunal activity [25].

Prediction models of the seafloor based on acoustic properties exist and provide reasonably good realizations of seafloor properties ([26] and references therein). But projections of acoustic reverberation, sediment characterization, and propagation often diverge from field observations because parameterizations underpinning operational models predominantly consider geophysical attributes of the system. From this perspective, seabed characteristics effectively equate with the physical substrate and ignore a more holistic view that combines substrate characteristics with the identity and the functional *modus operandi* of the resident fauna [27]. Indeed, acoustic systems with various operating frequencies are commonly used for the detection of objects buried in marine sediments. For example, buried wooden shipwrecks can be visualized using chirp signals with 1.5–13 kHz swept pulses [28], and the buried roots of aquatic plants with outer diameters of 5–10 cm have recently been imaged using ultrasound with a 100 kHz centre frequency [29]. Similarly, higher frequency signals, with a centre frequency of 1 MHz, can be used to survey bivalve molluscs (e.g. *Ruditapes philippinarum* [30]), while a centre frequency of 500 kHz has recently been used for the *in situ* detection of deep-sea infauna with calcified exoskeletons [31]. Nevertheless, the detection of buried objects using acoustics is impeded by the high attenuation of acoustic waves due to scattering and absorption in the sediment [15,16]. Consequently, there are only a few examples of benthic surveys that identify details below broad habitat types using acoustics [32] and, to date, it has not been possible to reliably identify invertebrate species directly from acoustic data. A logical next step would be to incorporate how biological communities modify seafloor sediment in models of seafloor processes [7], but progress in this area has been slow due to a lack of detailed mechanistic information about specific organism–sediment interactions.

Species, and traces of their activity, such as open or infilled burrows, can form discrete sources of acoustic scatter that can be readily distinguished from the surrounding sediment [30,33]. Here, we present and demonstrate the application of a three-dimensional acoustic ‘coring’ system capable of visualizing burrowing benthic organisms. We employ a three-dimensional convolutional neural network (3D-CNN) [34] to build a model that predicts the identity of species from species-specific backscatter contained within a local region of the sediment profile with an appropriate combination of data augmentation [35,36] and data correction techniques. Collectively, we show that combining appropriate acoustic systems and deep learning routines can provide detailed information about the presence, location and activity of subsurface benthic invertebrates.

## 2. Material and methods

### 2.1. Experimental set-up

**Figure 1.**
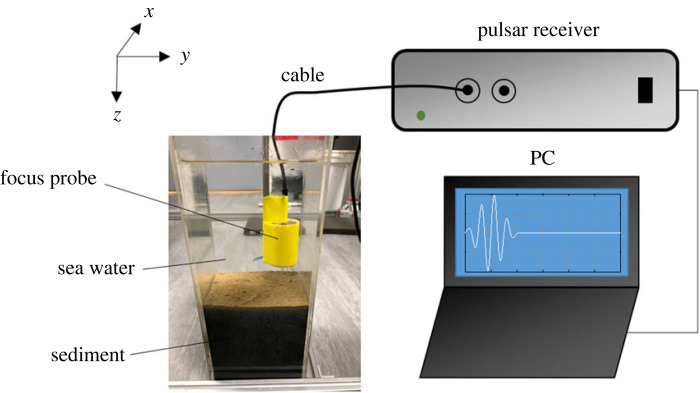
Automated acoustic measuring system used to determine the position and activity of macrofaunal invertebrates. The focused transducer probe is attached to a pole on the two-dimensional stage controlling unit, which is used to move the probe in *X* and *Y* directions (measurement area 6 × 6 cm^2^, at 0.1 cm intervals) to scan the sediment profile. The backscatter from individuals of the mud snail, *Peringia ulvae,* or clam, *Macoma balthica,* is recorded using a digital pulser receiver to generate two-dimensional and three-dimensional acoustic images based on waveforms.

Surficial sediment (less than 3 cm depth to avoid deeper sulfide enriched sediment: mean particle size, 54.80 µm; mud content, 55.93%) and two co-occurring functionally contrasting intertidal invertebrates (the gastropod *Peringia ulvae* (mud snail) and bivalve *Macoma balthica* (clam)) were collected from the mid-shore of the River Hamble, UK (50° 52′ 23.1″ N, 1° 18′ 49.3″ E) in June 2019. The sediment and organisms were returned to the Biodiversity and Ecosystem Futures Facility at the University of Southampton to acclimatize to laboratory conditions (5 days). Sediment was sieved (500 µm mesh) in a seawater bath (sand filtered, UV sterilized, salinity 33) to remove macrofauna, allowed to settle for 48 h to retain the fine fraction (less than 63 mm) and stirred to homogenize the distribution of sediment particles [37,38]. Each aquarium (internal dimensions, l × w × h: 12 × 12 × 35 cm^3^) was filled to a depth of 15 cm with sediment homogenate and overlain by 15 cm of seawater. Overlying seawater was replaced after 24 h to remove excess nutrients associated with assembly. Invertebrate fauna were introduced after 3 days, once a redox gradient was visible (coloration change, *sensu* [39]) and the lower regions of the cores showed evidence of reducing conditions (anoxic microniche formation, *sensu* [40]). Aquaria were maintained at 12 ± 0.1°C under a 12 : 12 h light–dark cycle (BioLumen, Aquabar T-series, Ultra Daylight spectra; Tropical Marine Centre, UK) and continually aerated. Replicate (*n* = 3) invertebrate communities were assembled in monoculture (the mud snail *Peringia ulvae*, PU; or the clam *Macoma balthica*, MB) with biomass fixed at 1 g wet weight (equivalent to approx. 150 ind. of PU or approx. 30 ind. of MB) in square acrylic aquaria. As our focus was to distinguish the acoustic signature of the fauna, and any modifications they make to the sedimentary environment, we also assembled replicate aquaria (*n* = 3) containing no macrofauna. Acoustic measurements were taken from all nine aquaria after 10 days (scan time approx. 10 min aquarium^−1^; figure 1, following [29]). Backscatter from the sediment and infaunal invertebrates is observed using an automatic measurement system (figure 1), comprising a two-dimensional waterproof stage control unit (custom-made, Arc Device, Koganei, Japan) and an acoustic measurement unit [30] held on a stage frame (l × w × h: 120 × 120 × 60 cm^3^) that holds the aquaria. Acoustic transducers are attached to the front edge of a pole extending from the moving stage, controlled using bespoke software generated in the Labview platform (National Instruments, Austin, TX). The moving step of the stage (precision, 0.01 cm in *X* and *Y* directions) varies from 0.1 cm to 100 cm. Backscatter is received by an acoustic focus probe (diameter = 3 cm, focal distance = 3.2 cm; Japan Probe, B1K25.4I PF38) and the square pulse, with a central frequency of 1 MHz, is generated by a pulser receiver (Japan Probe JPR-300C) and applied to the focus probe. Once programmed, the system automatically repeats stage positioning (moving and stopping) and acoustic measurements across the sediment surface (measurement area, 6 × 6 cm^2^, at 0.1 cm intervals). The distance between the probe and the sediment surface is maintained at approximately 5 cm. Backscatter is recorded using a digital pulser receiver with 10 MHz sampling rate, achieving 2000 recorded data points for a signal. The two-dimensional and three-dimensional acoustic images are generated on the basis of waveforms after the experiments. To detect the location of a species (i.e. individuals of *M. balthica* or *P. ulvae*) at or below the sediment surface, a three-dimensional acoustic image is constructed following the data processing flow developed by Mizuno *et al.* [29], setting the attenuation coefficient, *α*, to 50 dB m^−1^. In brief, to visualize the buried target, two-dimensional cross-sectional acoustic images at each depth were generated using scanned data for the *X*–*Y* plane based on ultrasonic C-mode imaging and a three-dimensional acoustic image (voxels) was reconstructed from these layers by stacking and alpha-blending processing. Finally, a smooth three-dimensional acoustic image of the space under the water bottom was constructed.

### 2.2. Three-dimensional convolutional neural network-based identification

**Figure 2.**
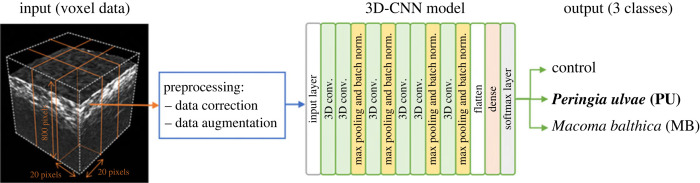
Overview of the 3D-CNN based prediction model used to determine the identity of two benthic organisms (gastropod, *Peringia ulvae*; bivalve, *Macoma balthica*) from three-dimensional acoustic returns. The measured three-dimensional data is divided into 20 × 20 × 800 voxel data, a volume appropriate to the size of the organisms, and used as input data for the 3D-CNN model. The details of the 3D-CNN model are shown in electronic supplementary material, figure S1.

To analyse the measured three-dimensional data, we constructed a classification model based on 3D-CNN [34,36] (figure 2). The acoustic image data are divided into 20 × 20 × 800 voxel data, which are then used by the 3D-CNN model to predict whether the acoustic image contains *P. ulvae*, *M. balthica*, or no fauna (control). The interval between pixels in the three-dimensional data is 1 mm for the *X* or *Y* direction and 75 µm for the *Z* direction. Here, the *Z* direction distance is converted by time × speed of sound (= *t* × 1500 m s^−1^). To capture information about the extension in the *Z* direction, we performed strong pooling in the *Z* direction. To investigate the effect of data preprocessing, we prepared the data with and without intensity correction (DC). Time varied gain (TVG) was performed to the original wave signal with 50 dB m in this study. To improve the learning performance, we performed data augmentation (DA) by shifting and flipping the voxel data [35,36]. For the shift, we shifted the data by up to 20% in the *X*, *Y* and *Z* directions.

### 2.3. Network training and evaluation

The training and evaluation of the network are based on a five-fold cross-validation, which divides the dataset into five parts, each used once as a test set and four times as training, to ensure thorough model evaluation. We trained the network using the Adam optimizer—an algorithm for first-order gradient-based optimization of stochastic objective functions, based on adaptive estimates of lower-order moments [41] with a learning rate of 0.0001 and a batch size of 2. Adam has the advantage that it is appropriate for non-stationary objects and very noisy and/or sparse gradients in signal. We determined prediction performance as the overall percentage of correct determinations of invertebrate species identity. In addition, to evaluate the prediction performance of the 3-class classification in a balanced manner, we calculated a macro F1 score as the overall evaluation and performed analysis using receiver operating characteristic (ROC) curve and area under the curve (AUC). The macro F1 score is the average of the F1 scores calculated for each class independently, where the F1 score for each class is the harmonic mean of precision and recall.

## 3. Results

### 3.1. Reconstructed three-dimensional acoustic images

**Figure 3.**
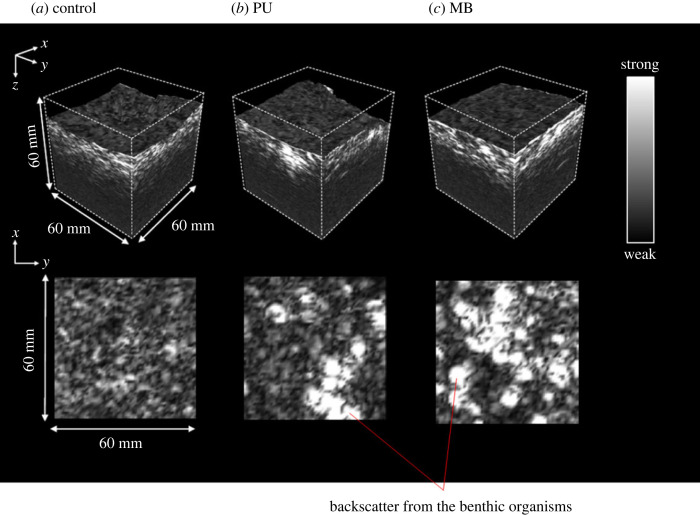
Three-dimensional (top) and two-dimensional (bottom) acoustic images to identify the subsurface presence of the mudsnail, *Peringia ulvae* (PU), and the clam, *Macoma balthica* (MB). Bottom row shows horizontal cross-sectional images 5 mm below the sediment surface. Lighter shading represents backscatter, signalling the presence of infaunal organisms and any associated sediment disturbance.

Three-dimensional acoustic images for the sediment volume below the sediment–water interface were reconstructed (voxel size 1 mm^3^; figure 3). Relative to the aquaria containing macrofauna, the aquaria containing no macrofauna had a less pronounced backscatter (to 5 cm depth; figure 3 *a*, figure 2). For *Peringia ulvae*, the scattered signals were vertically distributed to 4 cm sediment depth (figure 3 *b*; electronic supplementary material, figure S2), whilst scatter signals for *Macoma balthica* were generally concentrated within the uppermost 1 cm of the sediment profile (figure 3 *c*; electronic supplementary material, figure S2).

### 3.2. Identification of the benthic organisms

**Figure 4.**
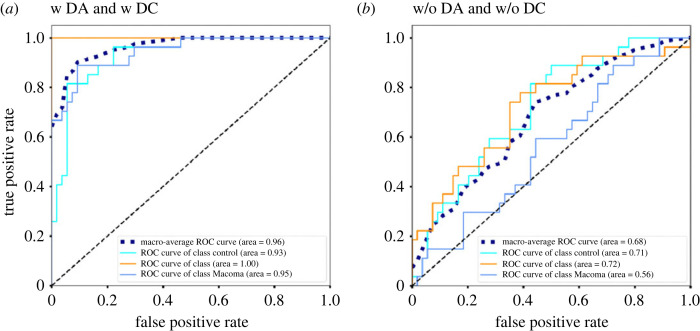
Evaluation of prediction performance of the 3D-CNN-based identification system (*a*) with and (*b*) without data augmentation (DA) and data correction (DC).

Using the 3D-CNN-based model, we evaluate the classification performance of three classes (control, PU and MB) based on five-fold cross-validation. We find that prediction accuracy is low without data collection (DC) and data augmentation (DA) pre-processing. However, when both DC and DA are used, accuracy substantially improves to 88%, with a macro AUC of 96% (table 1). ROC curves with and without both DC and DA reveal that the classification accuracy of *P. ulvae* is particularly high, whilst the no macrofauna control and *M. balthica* treatments are less discernable (figure 4). The latter suggests that prediction capability may be dependent on the morphology, burrowing depth and behaviour of individual species. The learning curves are shown in electronic supplementary material, figure S3.

**Table 1.** Summary of the accuracy of invertebrate identification, macro F1 score and macro area under the curve (AUC) offered by the 3D-CNN-based model with (w) and without (w/o) data augmentation (DA) and/or data correction (DC).

model	accuracy	macro F1 score	macro AUC
w/o DA and w/o DC	0.44	0.40	0.68
w DA and w/o DC	0.75	0.72	0.81
w/o DA and w DC	0.78	0.77	0.90
w DA and w DC	0.88	0.88	0.96

## 4. Discussion

Although numerous examples of biological and geophysical modification of the seafloor exist, their utility is often constrained to a particular setting or context [42] and the wide range of possible biological and physical interactions that can lead to non-additive effects are not generally considered. Here, using the acoustic properties of sediments containing known species and their associated biogenic structures, we have been able to demonstrate that species can provide sufficiently distinctive acoustic responses that can be recognized using deep learning methods. Should these findings hold true across a broader range of species, our approach allows acoustically contrasting groups (e.g. tube builders, burrowers, soft versus hard body parts) within representative sediment types (e.g. sand, muddy-sand, sandy-mud, mud [43]) to be used to establish generality, which can then be applied to untested context-dependent biodiversity scenarios [44] and/or inform models attempting to form a comparative view of seabed functioning [7,27].

The presented approach is designed to capture acoustic responses from the seafloor, that is, the acoustic reflection and scattering from the sediment profile which, in turn, will be highly dependent on the source, composition and stability of the sediment and the biomass, composition and functional diversity of macroinvertebrates. Species can also dynamically alter their behaviour in response to a wide range of abiotic (e.g. [45,46]) and biotic (e.g. predation [47]) circumstances, which may vary with season, abiotic or biotic setting [42,48] and/or climate forcing [38]. Rather than confound observations of species–sediment interactions, a strength of our approach is that these sources of variation can be explicitly embraced as the presented methodology is non-invasive and can be extended to track species’ (or individuals’) responses to gradual change with outcomes used to inform the deep learning algorithms. Our expectation is that prediction accuracy may decrease if multiple species or large shifts in density occur in a natural assemblage, but we anticipate that predictive capacity will improve to acceptable levels with further training of the machine learning algorithms. This raises the prospect of being able to use deep learning algorithms to non-invasively quantify the functional contribution of individuals and species within an intact community as they respond to changing circumstances [49] or predict future ecological consequences of altered biodiversity within the context of a dynamic system [44].

## 5. Conclusion

Benthic species–sediment relations are fundamental to the mediation of many ecosystem processes and properties, yet mechanistic understanding has been frustrated by limited capabilities in *in situ* subsurface observation methods. The acoustic corer system presented here provides a method that can be used in laboratory and field settings to non-invasively determine the presence, location and activity of subsurface benthic invertebrates from acoustic backscatter using deep learning algorithms. Sensor installation is straightforward and requires a fixed position within the vicinity of the sediment–water interface under study, and data acquisition is sufficiently brief that the methodology can be used in highly replicated experiments. We demonstrated the utility of the system with two co-occurring, but functionally contrasting, macrofaunal species, and for a sediment that contained no macrofauna, and achieved an acceptable level of accuracy. This new capability presents the possibility of identifying which, and when, individuals and/or species are most active in dynamic naturally assembled communities [29,31].

## Data Availability

The code for the machine learning is available on GitHub at https://github.com/ycu-iil/Acoustic3DCNN. These data have been archived on Zenodo at https://doi.org/10.5281/zenodo.11054876 [50]. Supplementary material is available online [51].
